# Low-fibre diet is associated with high-risk coronary plaque features

**DOI:** 10.1093/cvr/cvaf088

**Published:** 2025-06-16

**Authors:** Ingrid Larsson, Jiangming Sun, Shafqat Ahmad, Göran Bergström, Carl-Johan Carlhäll, Kerstin Cederlund, Isabel Drake, Jan E Engvall, Mats Eriksson, Henrik Hagström, Tomas Jernberg, Tanja Kero, Krister Lindmark, Maria Mannila, Marju Orho-Melander, Araz Rawshani, Ulf Risérus, Annika Rosengren, Mats Ryberg, Caroline Schmidt, Emily Sonestedt, Maria Wennberg, Carl Johan Östgren, Isabel Goncalves

**Affiliations:** Department of Molecular and Clinical Medicine, Institute of Medicine, Sahlgrenska Academy, University of Gothenburg, Gothenburg, Sweden; Department of Medicine, Sahlgrenska University Hospital, Gothenburg, Sweden; Cardiovascular Research Translational Studies, Department of Clinical Sciences Malmö, Lund University, Malmö, Sweden; Molecular Epidemiology and Science for Life Laboratory, Department of Medical Sciences, Uppsala University, Uppsala, Sweden; Preventive Medicine Division, Harvard Medical School, Brigham and Women’s Hospital, Boston, USA; School of Natural Sciences, Technology and Environmental Studies, Södertörn University, Stockholm, Sweden; Department of Molecular and Clinical Medicine, Institute of Medicine, Sahlgrenska Academy, University of Gothenburg, Gothenburg, Sweden; Clinical Physiology, Sahlgrenska University Hospital, Gothenburg, Sweden; Department of Clinical Physiology in Linköping, Linköping University, Linköping, Sweden; Department of Health, Medicine and Caring Sciences, Linköping University, Linköping, Sweden; Center for Medical Image Science and Visualization, Linköping University, Linköping, Sweden; Department of Clinical Science, Intervention and Technology, Karolinska Institutet, Stockholm, Sweden; Department of Clinical Sciences in Malmö, Lund University, Malmö, Sweden; Clinical Physiology, Sahlgrenska University Hospital, Gothenburg, Sweden; Department of Clinical Physiology in Linköping, Linköping University, Linköping, Sweden; Department of Health, Medicine and Caring Sciences, Linköping University, Linköping, Sweden; Department of Endocrinology, Karolinska University Hospital, Stockholm, Sweden; Department of Public Health and Clinical Medicine and Heart Centre, Umeå University Hospital, Umeå University, Umeå, Sweden; Department of Clinical Sciences, Danderyd University Hospital, Karolinska Institutet, Stockholm, Sweden; Medical Imaging Centre, Uppsala University Hospital, Uppsala, Sweden; Department of Surgical Sciences/Radiology, Uppsala University, Uppsala, Sweden; Department of Clinical Sciences, Danderyd University Hospital, Karolinska Institutet, Stockholm, Sweden; Department of Public Health and Clinical Medicine, Umeå University, Umeå, Sweden; Department of Cardiology and Clinical Genetics, Karolinska University Hospital, Stockholm, Sweden; Department of Clinical Sciences in Malmö, Lund University, Malmö, Sweden; Department of Molecular and Clinical Medicine, Institute of Medicine, Sahlgrenska Academy, University of Gothenburg, Gothenburg, Sweden; Department of Cardiology, Sahlgrenska University Hospital, Gothenburg, Sweden; Clinical Nutrition and Metabolism, Department of Public Health and Caring Sciences, Uppsala University, Uppsala, Sweden; Department of Molecular and Clinical Medicine, Institute of Medicine, Sahlgrenska Academy, University of Gothenburg, Gothenburg, Sweden; Department of Medicine Geriatrics and Emergency Medicine, Sahlgrenska University Hospital Östra Hospital, Gothenburg, Sweden; Department of Public Health and Clinical Medicine, Umeå University, Umeå, Sweden; Department of Molecular and Clinical Medicine, Institute of Medicine, Sahlgrenska Academy, University of Gothenburg, Gothenburg, Sweden; Department of Clinical Sciences in Malmö, Lund University, Malmö, Sweden; Department of Public Health and Clinical Medicine, Section of Sustainable Health, Umeå University, Umeå, Sweden; Department of Health, Medicine and Caring Sciences, Linköping University, Linköping, Sweden; Center for Medical Image Science and Visualization, Linköping University, Linköping, Sweden; Cardiovascular Research Translational Studies, Department of Clinical Sciences Malmö, Lund University, Malmö, Sweden; Department of Cardiology, Skåne University Hospital, Jan Waldenströms Gata 35, 91-12, Malmö S-20502, Sweden

**Keywords:** Anti-inflammatory, Diet, Cardiovascular disease, Coronary plaque, Stenosis

## Abstract

**Aims:**

Diet is a determinant of cardiovascular diseases (CVD) with coronary disease as predominant cause of pre-mature death. To analyse how diet was associated with coronary atherosclerosis, including plaque features.

**Methods and results:**

The cross-sectional population-based study using data from the Swedish CArdioPulmonary BioImage Study (SCAPIS) included 24 079 adults aged 50–64 years, recruited in 2013 to 2018 who were free of clinical cardiovascular disease. The recruitment and comprehensive examinations were conducted at six locations in Sweden. A dietary index (DI) based on a previously published anti-inflammatory DI including high proportion of plant-based foods, and low in red or processed meat and sugar-sweetened beverages was constructed. The reference group was within lowest DI tertile. Coronary atherosclerosis assessed by coronary computed tomography angiography, including any-, significant-, and adverse or high-risk coronary plaque, which is non-calcified with a significant stenosis ≥50%. Lowest, compared to highest DI tertile was associated with younger age, more often men (62.2% vs. 32.9%), higher high-sensitive C-reactive protein, more cardiometabolic risk and smokers, higher alcohol-, and higher energy-intake. In the highest and lowest tertile, coronary plaques were present in 36.3% and 44.3%, respectively, stenosis ≥ 50% in 3.7% and 6.0%. Non-calcified coronary plaques with stenosis ≥50% were present in 0.9% and 1.5% in highest and lowest tertiles. In multivariable analyses, the lowest tertile of DI was associated with high-risk plaque features after adjusting for age, sex, smoking, with waist circumference, triglycerides (TGs), and hypertension as possible mediators.

**Conclusion:**

A low-fibre diet with high red meat content was associated with high-risk plaques features, increased coronary calcification and significant stenosis. Waist circumference, TGs, and hypertension emerged as potential mediators of these associations, underscoring the role of metabolic and hemodynamic factors in the dietary impact on coronary atherosclerosis. Our findings strengthen the importance of cardioprotective dietary recommendations.

## Graphical Abstract

**Graphical Abstract:**
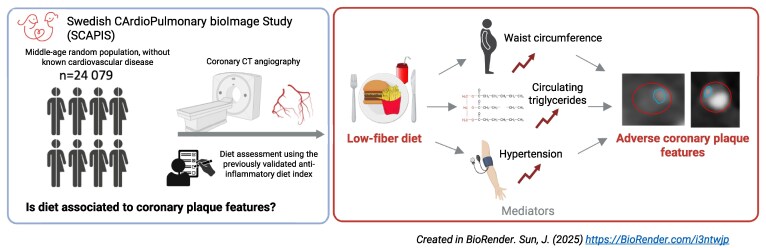

**Time of primary review: 49 days**

**See the editorial comment for this article ‘*Let food be thy medicine:* anti-inflammatory diets and the hidden properties of coronary plaque’, by E. Jones and T. J. Guzik, https://doi.org/10.1093/cvr/cvaf115.**

## 1. Introduction

Diet is an important determinant of disease.^1^ The Mediterranean diet and similar patterns^2^ have been associated with lower risk of cardiovascular morbidity^3^ and mortality.^2,4,5^ Different dietary indices have identified patterns, such as the Mediterranean and other high-fibre diets, as likely to be associated with lower risk of disease.^5^ Cardiovascular disease (CVD) remains the most important cause of death and disability,^6^ with coronary disease a predominant cause of pre-mature death. The most common underlying pathology is atherosclerosis, where plaques are formed along decades, with some prone to rupture,^7^ causing myocardial infarction, or sudden coronary death. However, the occurrence of coronary events is not only associated with the degree of stenosis caused by a plaque, but also with plaque composition.^8^ Still, accessible non-invasive diagnostic methods to detect rupture-prone plaques have been lacking. In recent years, coronary computed tomography angiography (CCTA) has emerged as promising and is recommended in international guidelines for risk stratification for low/moderate risk patients.^9,10^ Plaques with adverse or high-risk features, for example low attenuation on CCTA in combination with significant stenosis, have been linked to a higher risk for cardiovascular events.^11,12^

Dietary patterns that include a high intake of vegetables, fruits, and whole-grain cereals are associated with lower risk of CVD.^1,3,13^ Dietary indices have been used as composite variables constructed from food items known *à priori* to be healthy, alternatively unhealthy.^5,13^ Kaluza *et al*.^14^ identified food items from a food frequency questionnaire (FFQ), that correlated to indices of inflammation such as elevated levels of high sensitivity C-reactive protein (hsCRP) and grouped them into a diet index (DI) including foods with anti-inflammatory potential (fruits and vegetables, nuts, whole-grain bread, breakfast cereals and oat-meal, low-fat cheese, canola- and olive-oil, coffee, tea, chocolate, red wine, and beer) and foods with pro-inflammatory potential, (unprocessed and processed red meat, offal, potato chips, and sugar-sweetened beverages).^14^ In Swedish prospective cohort studies, men and women with high DI and low intake of foods with pro-inflammatory potential had lower risk of mortality,^15^ abdominal aortic aneurysm,^16^ and heart failure^17^ after 16 years follow-up, compared to those with low DI. Using isotope-ratio mass spectrometry, it was demonstrated that dietary intake is reflected in rupture-prone plaque components.^18^ However, potential associations between diet intake and the presence of coronary artery plaque, or their features, in CCTA remain unexplored in large population-based cohorts.

The aim of this study was to analyse whether diet intake assessed by a DI based on the anti-inflammatory DI by Kaluza *et al*.^14^ is associated with coronary atherosclerosis, coronary plaque features assessed by CCTA and known cardiovascular risk factors, in a randomly selected, population-based cohort of middle-aged Swedish subjects. The hypothesis was that lower intake of fibre-rich components and higher intake of red and processed meat in the diet was associated with more adverse or high-risk coronary plaque features.

## 2. Methods

### 2.1 Study population

The Swedish CArdioPulmonary BioImage Study (SCAPIS) is a population-based study that included 30 154 (male and female) participants (aged 50 to 64 years) from randomly selected individuals at six Swedish sites.^19^ Based on the unique Swedish personal identification number, an unbiased and randomized selection was performed from the Swedish population register. An information brochure to those selected was sent by letter, with an invitation to contact the research team if they were interested in participating in the study. Recruitment from the selected group was reinforced by advertisements in newspapers and on television. Overall participation rate was 50%.^19^

Besides the previously described SCAPIS study design, in the present study the following subjects were excluded: (i) incomplete or extreme values of dietary data, (ii) unsuccessful CCTA (for technical reasons), and (iii) previous known CVD or presence of stent in CCTA. No other exclusion criteria were used in the selection. Out of 30 154 subjects from SCAPIS, 24 079 (80%) were used in the present study. Like in the larger SCAPIS, sex is balanced with 51% women and 49% men, median aged 57 (54–61) years old.

**Figure 1:**
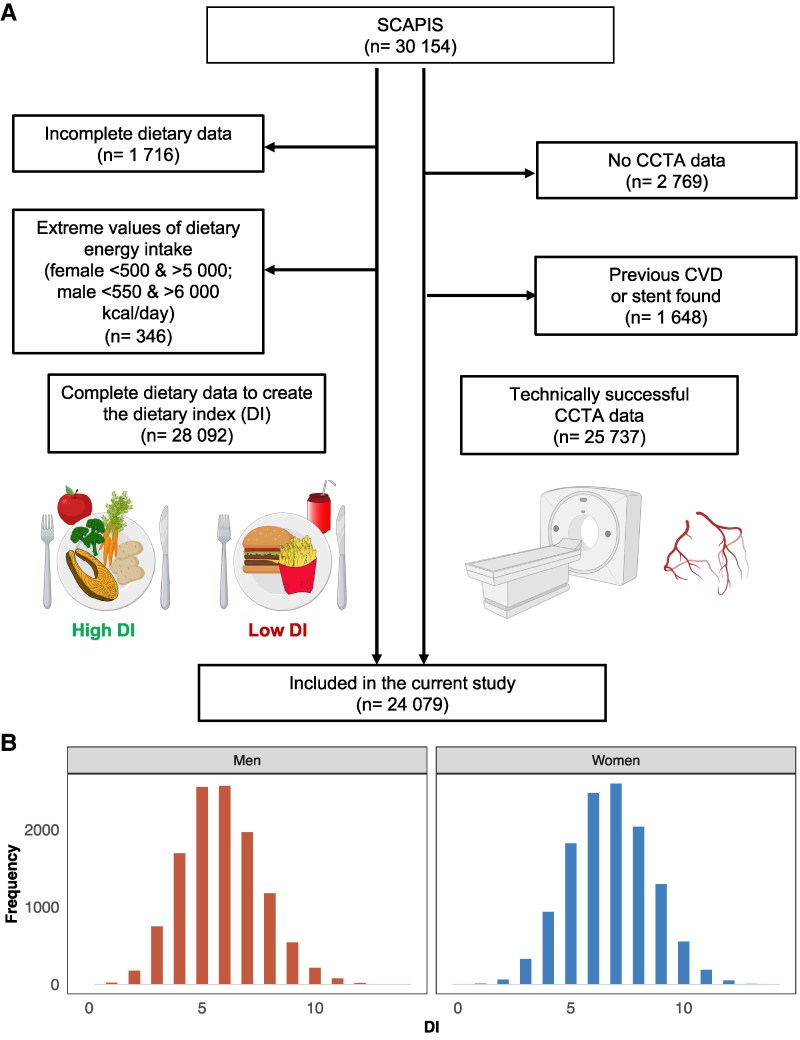
(*A*) Flow diagram of study inclusion and exclusion, created in BioRender. Sun, J. (2025) https://BioRender.com/i3ntwjp. 24 079 participants are included in this study. (*B*) Distribution of the DI in women (*n* = 12 340) and men (*n* = 11 739).

To assess coronary atherosclerosis CCTA was performed. In *Figure 1A*, the flowchart of the population in this cross-sectional study is shown. The cardiovascular risk factors were registered and are described in Supplementary material online, *Table S1*.

### 2.2 Ethical declaration

The multi-centre study was approved by the ethical review board in Umeå (2010-228-31M).^20^ All participants gave informed consent prior to the inclusion in the study. The study conformed to the principles outlined in the Declaration of Helsinki.

### 2.3 Dietary assessment

The MiniMeal-Q is a web-based, self-administered, semi-quantitative FFQ including 75 to 126 single food items and mixed dishes, depending on food selection, covering habitual intake during the past few months (see Supplementary material online, Materials).^21^ The composition of the DI is presented in *Table 1*. If a cut-off for serving was met, score 1 was given, otherwise score 0. In total, 11 foods and beverages had anti-inflammatory potential, and five foods and beverages had pro-inflammatory potential, as described above (*Table 1*). The score ranged from 0 (none of the cut-offs were met) to 16 (all cut-offs were met). Higher DI means higher reported intake of fibres, fruits and vegetables, nuts, whole-grain bread, breakfast cereals and oat-meal porridge, low-fat cheese, canola- and olive-oil, coffee, tea, red wine, beer, and lower reported intake of unprocessed red meat and processed red meat, offal, potato chips, and sugar-sweetened beverages.

**Table 1:** Description of foods with anti-inflammatory and pro-inflammatory potential used to construct a DI

	Amount per serving	Reported consumption for score 1, otherwise score 0^a^	Number of the participants fulfilling the criterion for each food item
Anti-inflammatory potential			
Total fruits and vegetables	75 g	≥6 per day	3362 (14.0%)
Nuts	28 g	≥3 per week	7659 (31.8%)
Whole-grain bread including hard crisp bread	70 g	≥2 per day	3244 (13.5%)
Breakfast cereals and oat-meal porridge	50 g	≥1 per day	5731 (23.8%)
Low-fat cheese	15 g	≥1 per day	11 876 (49.3%)
Olive- and canola oil	15 mL	>0 per day	19 961 (82.9%)
Coffee	150 mL	≥2 per day	18 992 (78.9%)
Tea	250 mL	≥3 per day	1702 (7.1%)
Chocolate	28 g	≥1 per day	1371 (5.7%)
Red wine	120 mL	2–7 per week	2653 (11.0%)
Beer, ≤ 3.5 vol%	330–500 mL	2–14 per week	450 (1.9%)
Pro-inflammatory potential			
Unprocessed red meat	125 g	≤0.5 per day	23 345 (96.9%)
Processed red meat	100 g	≤0.5 per day	13 632 (56.6%)
Offal		0 per day	18 932 (78.6%)
Potato chips or cheese doodles		0 per day	10 202 (42.4%)
Sugar-sweetened soft drinks		0 per day	8669 (36.0%)

^a^The scores were developed according to Kaluza *et al*.^15^

### 2.4 Coronary atherosclerosis plaques assessment

The collection and interpretation of CCTA, number of segments involved, and coronary artery calcification (coronary artery calcium score, CACS) has been described in other studies.^22,23^

Six different CAD outcomes were analysed: (i) any coronary atherosclerosis; (ii) segment involvement score (SIS) ≥ 4 (indicating high coronary atherosclerotic burden); (iii) CACS >100 Agatston units (indicating highly calcified atherosclerosis); (iv) any significant stenosis (≥50%); (v) any non-calcified plaque (often called low-attenuating plaque); (vi) plaque phenotypes according to the Scottish COmputed Tomography of the HEART Trial (SCOT HEART), considering plaques calcified with stenosis <50%, non-calcified and stenosis <50%, calcified plaques and stenosis ≥50%, and finally non-calcified and stenosis ≥50%.^11^

A plaque was defined as a visual entity ≥1 mm^2^ within the vessel wall, and clearly distinctive from tissue surrounding the vessel and the vessel lumen; a low attenuating plaque was a plaque without any visible calcification.^24^

The study involved 36 trained readers with 1 to over 10 years of experience in interpreting CCTA (no core lab). The readers were categorized into three competence levels: level 1 (11 readers, 11% of all readings), level 2 (16 readers, 55.6%), and level 3 (9 readers, 33.4%). They visually interpreted CCTA datasets and entered their findings into an electronic case report form. To ensure consistent reporting, readers participated in yearly training sessions. To assess the reproducibility of CCTA readings, two readers independently evaluated 51 consecutive cases more than four weeks apart. The consistency of data across readers and sites in detail has been previously published.^20^ To improve efficiency and focus on the most clinically significant findings, readers prioritized reporting on 11 key mandatory coronary segments (segments 1–3, 5–7, 9, 11–13, and 17). Other segments were only reported if they showed atherosclerosis or calcium blooming. Using syngo via CT (Siemens Healthineers, Forchheim, Germany), each coronary segment was visually evaluated for the presence of plaques, which were categorized as either calcified or non-calcified. The terms ‘only non-calcified plaques’ and ‘any plaque non-calcified’ were used to describe participants with exclusively non-calcified plaques or a combination of non-calcified and calcified plaques, respectively.^19,20^ The density/attenuation of non-calcified plaques was not measured. The status of each coronary vessel was classified per segment as: no atherosclerosis, 1%–49% stenosis, ≥50% stenosis (significant stenosis), not assessable due to calcium blooming, or not assessable due to technical issues or missing data. Luminal obstruction was assessed by visually estimating the diameter stenosis, using the average of the longest and shortest diameters at the site of the stenosis.

Various protocols were used depending on the heart rate, the regularity of the heart rate and body weight as previously described,^19^ with 0.27% having used the arrhythmia protocol, though no information was obtained to allow the exact diagnosis underlying the irregularity of the heart rate.

All examinations were performed on 2 or 3 occasions within a 2-week period.^19^ The questionnaires were completed by the participants at one of these occasions.^19^

### 2.5 Statistical analysis

Characteristics of participants were compared by tertiles of DI using the Kruskal–Wallis test for continuous variables and χ^2^ tests for categorical variables.

To evaluate variable importance in the association, multinomial and binary logistic regressions were performed using the following variables as possible predictors: age, sex, smoking, waist circumference (WC), triglyceride (TG), hsCRP, estimated glomerular filtration rate (eGFR), moderate to vigorous physical activity (MVPA), sleep hours, alcohol intake, stress feelings, energy intake, education, born in Sweden, employment and presence of hypertension, hyperlipidaemia, diabetes, and family history of myocardial infarction or stroke. The analysis was performed iteratively removing one variable at a time. Change in *R*^2^ when removing one variable from the regression was used to rank the variable importance. This data-driven strategy was used to select the variables included in the adjustment models below.

Associations between DI (categorized into tertiles) and plaque phenotypes were examined using multinomial logistic regression and binary logistic regression.

Five models were implemented: (i) crude; (ii) adjusted for age and sex to achieve minimal adjustment; (iii) model 2 further adjusted for smoking without potential mediators; (iv) model 3 further adjusted for potential mediators, including WC, hypertension, and TG; and (v) adjusted for all the 19 studied factors.

Using DI of 8–14 (highest tertile) as reference, odds ratios (ORs) were reported for DI of 6–7 (middle tertile) and 0–5 (lowest tertile), respectively. The interaction between DI tertiles and sex in relation to plaque phenotypes was evaluated, adjusting for age. Mediation analysis was performed for WC, hypertension, and TG (see Supplementary material online, Materials), as these factors are known to be influenced by dietary habits^13,25^ and in turn, serve as key risk factors for plaque burden.^25^

## 3. Results

From the 30 154 SCAPIS participants, 79.9% (*n* = 24 079) have been included in the present study with concomitant dietary data allowing the assessment of DI and technically successful CCTA data (*Figure 1A*). Details regarding the excluded patients are described in Supplementary material online, Material. The distribution of the DI values in men and women is presented in *Figure 1B*.

### 3.1 DI, clinical characteristics, and coronary atherosclerosis

The range of DI was 0 to 14 in the subjects. Characteristics by DI tertile intake are presented in *Table 2* (characteristics in relation to the presence of coronary plaque are presented in Supplementary material online, *Table S2*). Subjects in the lowest compared to the highest DI tertile had higher hsCRP, were more often men (62.2% vs. 32.9%), slightly younger, with less family history of cardiovascular events, larger WC, more current smoking and alcohol intake, more hyperlipidaemia, hypertension, and diabetes, were less educated and spent less time in MVPA. The presence of any plaque in CCTA was higher in subjects in the lowest DI tertile and accordingly, with a less anti-inflammatory dietary pattern. Similarly, these subjects had more calcified than non-calcified plaques, higher CACS, more segments involved, and more significant stenoses (≥ 50%). Finally, participants in the lowest compared to the highest DI tertile also had more high-risk plaque features (low attenuation and significant stenosis), although this pattern was uncommon (*Table 2*).

**Table 2:** Characteristics of the SCAPIS population in relation to the DI

	Total (*n* = 24 079)	T1 (*n* = 8344)	T2 (*n* = 9596)	T3 (*n* = 6139)	*P*-value
Coronary atherosclerotic burden					
Any coronary plaque					<0.001
No	14 245 (59.2%)	4651 (55.7%)	5684 (59.2%)	3910 (63.7%)	
Yes	9834 (40.8%)	3693 (44.3%)	3912 (40.8%)	2229 (36.3%)	
Calcified coronary plaque					<0.001
No plaque	14 245 (59.2%)	4651 (55.7%)	5684 (59.2%)	3910 (63.7%)	
Plaque non-calcified	1776 (7.4%)	714 (8.6%)	671 (7.0%)	391 (6.4%)	
Plaque calcified	8058 (33.5%)	2979 (35.7%)	3241 (33.8%)	1838 (29.9%)	
Significant coronary plaque					<0.001
No plaque	14 245 (59.2%)	4651 (55.7%)	5684 (59.2%)	3910 (63.7%)	
Stenosis <50%	8600 (35.7%)	3196 (38.3%)	3402 (35.5%)	2002 (32.6%)	
Stenosis ≥50%	1234 (5.1%)	497 (6.0%)	510 (5.3%)	227 (3.7%)	
SIS (≥4 segments)					<0.001
No	22 025 (91.5%)	7488 (89.7%)	8781 (91.5%)	5756 (93.8%)	
Yes	2054 (8.5%)	856 (10.3%)	815 (8.5%)	383 (6.2%)	
Total CACS					<0.001
≤100	21 185 (88.6%)	7173 (86.6%)	8468 (88.8%)	5544 (90.9%)	
>100	2732 (11.4%)	1107 (13.4%)	1071 (11.2%)	554 (9.1%)	
SCOT HEART					<0.001
No plaque	14 245 (59.2%)	4651 (55.7%)	5684 (59.2%)	3910 (63.7%)	
Non-calcified and stenosis <50%	1475 (6.1%)	585 (7.0%)	554 (5.8%)	336 (5.5%)	
Calcified and stenosis <50%	7125 (29.6%)	2611 (31.3%)	2848 (29.7%)	1666 (27.1%)	
Non-calcified, stenosis ≥ 50%	301 (1.3%)	129 (1.5%)	117 (1.2%)	55 (0.9%)	
Calcified and stenosis ≥ 50%	933 (3.9%)	368 (4.4%)	393 (4.1%)	172 (2.8%)	
Risk factors					
Sex					<0.001
Women	12 340 (51.2%)	3156 (37.8%)	5066 (52.8%)	4118 (67.1%)	
Men	11 739 (48.8%)	5188 (62.2%)	4530 (47.2%)	2021 (32.9%)	
Age (years)	57.1 (53.5, 61.0)	56.3 (52.8, 60.3)	57.2 (53.6, 60.9)	58.3 (54.4, 61.8)	<0.001
hsCRP (mg/L)	1.0 (0.60, 2.1)	1.2 (0.60, 2.4)	1.0 (0.60, 2.1)	0.80 (0.60, 1.7)	<0.001
Family history of MI or stroke, subject’s parent, or sibling					<0.001
No	12 859 (55.9%)	4652 (58.2%)	5062 (55.2%)	3172 (53.8%)	
Yes	10 159 (44.1%)	3327 (41.8%)	4110 (44.8%)	2722 (46.2%)	
Hyperlipidaemia or statin use					<0.001
No	21 787 (90.5%)	7479 (89.6%)	8660 (90.2%)	5648 (92.0%)	
Yes	2292 (9.5%)	865 (10.4%)	936 (9.8%)	491 (8.0%)	
Hypertension, doctor-diagnosed, or self-reported					<0.001
No	18 592 (9.8%)	6144 (76.4&)	7459 (80.1%)	4989 (83.7%)	
Yes	4716 (20.2%)	1894 (23.6%)	1851 (19.9%)	971 (16.3%)	
Diabetes					<0.001
No	22 556 (93.8%)	7727 (92.7%)	8990 (93.8%)	5839 (95.2%)	
Yes	1499 (6.2%)	607 (7.3%)	598 (6.2%)	294 (4.8%)	
Waist circumference (cm)	94.0 (85.0, 102.0)	97.0 (89.0, 105.0)	93.0 (85.0, 101.0)	88.0 (81.0, 97.0)	<0.001
Smoking, snuff, or nicotine products use					<0.001
Current	5466 (23.1%)	2433 (29.7%)	2122 (22.5%)	911 (15.1%)	
Ex-smoker	7567 (32.0)	2295 (28.0%)	3134 (33.2%)	2138 (35.4%)	
Never	10 627 (44.9%)	3457 (42.2%)	4185 (44.3%)	2985 (49.5%)	
MVPA, minutes per day	52.6 (36.3, 72.1)	50.5 (34.3, 70.9)	52.0 (36.0, 70.6)	56.1 (39.4, 75.8)	<0.001
Stressed or feelings of sadness/depression in the past 1 year					0.002
Less stressed and no feelings of sadness/depression	14 901 (64.4%)	5274 (65.9%)	5863 (63.6%)	3764 (63.5%)	
Stressed, or feelings of sadness/depression	8246 (35.6%)	2726 (34.1%)	3352 (36.4%)	2168 (36.5%)	
Quality of sleep					0.628
Badly–very badly	3732 (16.0%)	1285 (16.0%)	1470 (15.8%)	977 (16.4%)	
Rather well–very well	19 571 (84.0%)	6757 (84.0%)	7830 (84.2%)	4984 (83.6%)	
Hours of sleep per night					<0.001
7 h	10 417 (44.8%)	3421 (42.6%)	4199 (45.3%)	2797 (47.0%)	
≤6 h	8402 (36.1%)	3152 (39.3%)	3278 (35.4%)	1972 (33.1%)	
≥8 h	4424 (19.0%)	1451 (18.1%)	1792 (19.3%)	1181 (19.8%)	
Sleep apnoea treated					<0.001
No	22 630 (97.1%)	7756 (96.6%)	9045 (97.2%)	5829 (97.8%)	
Yes	664 (2.9%)	275 (3.4%)	259 (2.8%)	130 (2.2%)	
Education level					<0.001
Undergraduate degree or above	10 953 (46.6%)	2917 (35.9%)	4493 (47.9%)	3543 (59.0%)	
Up to upper secondary school or equivalent	12 569 (53.4%)	5214 (64.1%)	4895 (52.1%)	2460 (41.0%)	
Born in Sweden					<0.001
No	3511 (14.9%)	970 (11.9%)	1473 (15.7%)	1068 (17.8%)	
Yes	20 027 (85.1%)	7164 (88.1%)	7926 (84.3%)	4937 (82.2%)	
Employment					<0.001
No	3284 (14.0%)	850 (14.2%)	1269 (13.5%)	1165 (14.4%)	
Yes	20 177 (86.0%)	5140 (85.8%)	8100 (86.5%)	6937 (85.6%)	
Cardiometabolic and renal blood markers					
TG (mmol/L)	1.0 (0.8, 1.4)	1.2 (0.8, 1.6)	1.0 (0.8, 1.4)	0.9 (0.7, 1.2)	<0.001
Cholesterol (mmol/L)	5.5 (4.8, 6.2)	5.4 (4.8, 6.1)	5.5 (4.8, 6.2)	5.5 (4.9, 6.2)	<0.001
HDL (mmol/L)	1.6 (1.3, 1.9)	1.4 (1.2, 1.8)	1.6 (1.3, 1.9)	1.7 (1.4, 2.1)	<0.001
LDL (mmol/L)	3.4 (2.8, 4.1)	3.4 (2.8, 4.1)	3.4 (2.8, 4.1)	3.4 (2.8, 4.0)	0.013
eGFR (mL/min per 1.73 m^2^)	99.9 (90.0, 104.9)	98.4 (87.1, 104.2)	100.1 (90.4, 105.2)	101.1 (95.0, 105.5)	<0.001
Dietary variables					
Energy intake (kcal/day)	1585 (1240, 2035)	1527 (1197, 1957)	1540 (1198, 1981)	1737 (1384, 2213)	<0.001
Alcohol (g/day)	6.0 (2.0, 10.9)	6.5 (2.5, 11.9)	5.9 (2.0, 10.6)	5.4 (1.8, 9.8)	<0.001

All variables are given in number (%) or median (inter-quartile range), if not stated otherwise. *P*-values are from ANOVA for continuous variables or χ^2^ test for categorical variables.

T1, lowest tertile of DI 0–5; T2, middle tertile of DI 6–7; T3, highest tertile of DI 8–14; CACS, coronary artery calcium score; MVPA, moderate-vigorous-intensity physical activity; SCOT HEART, plaque phenotypes according to the Scottish COmputed Tomography of the HEART Trial.

A nominally significant interaction effect between DI and sex was found in predicting the presence of coronary plaque, particularly stronger in women than in men (see Supplementary material online, *Table S3*). No other significant interactions between DI and sex were observed in predicting the other plaque phenotypes.

**Figure 2:**
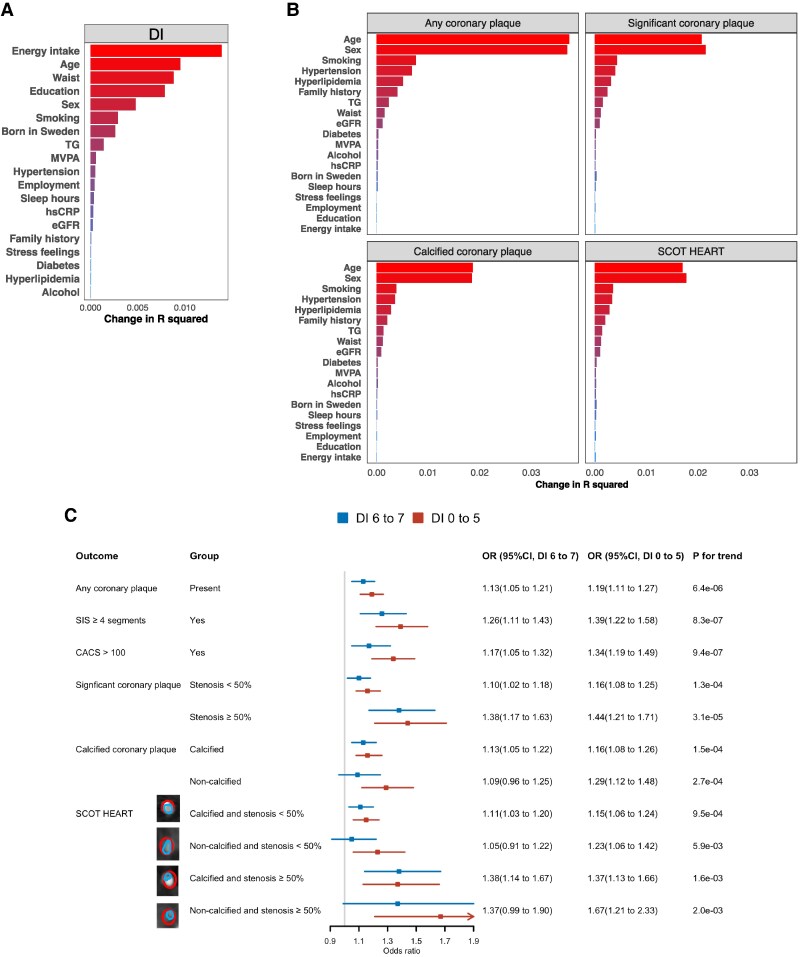
Factors associated with (*A*) the DI and (*B*) plaque phenotypes. 24 079 participants are included in the analyses. Multinomial logistic regression and binary logistic regression are performed using age, sex, smoking, waist circumference, TG, hsCRP, eGFR, MVPA, sleep hours, alcohol intake, stress feelings, energy intake, education level, born in Sweden, employment and presence of hypertension, hyperlipidaemia, diabetes, and family history of myocardial infarction or stroke as predictors. Multinomial logistic regression and binary logistic regression are also performed when iteratively removing one variable out. Changes in *R*^2^ are then obtained by comparing McFadden *R*^2^ (multinomial logistic regression) or Nagelkerke *R*^2^ (logistic regression) from the above model with all listed factors and to leave-one-variable-out to evaluate variable importance. (*C*) Age- and sex-adjusted OR for association between DI and plaque phenotype, showing representative cross-sectional images of each phenotype by CCTAs (vessel with plaque outlined in red and lumen in blue). 24 079 participants are used in the analysis. SIS, segment involvement score. SIS represents the total number of coronary segments with plaque. CACS, coronary artery calcium score; CCTA, coronary computed tomography angiography; SCOT HEART, plaque phenotypes according to the Scottish COmputed Tomography of the HEART Trial.

The associations between known cardiovascular risk factors and diet intake, as well as the four main coronary outcomes are presented in *Figure 2*. Energy intake and education level contributed more to the variance of the DI (*Figure 2A*) but had lower contributions to plaque presence and their characteristics (*Figure 2B*). Age and sex were the most important variables contributing to all plaque outcomes (*Figure 2B*). Age, sex, smoking, WC, TG, and hypertension were associated with both DI and plaque outcomes. No strong correlations between them were observed (see Supplementary material online, *Figure S1*), except women had higher eGFR, lower WC and reported less alcohol intake than men.

### 3.2 Association between DI and coronary atherosclerosis

Adjusting for age and sex, the lowest and middle tertiles of DI were significantly associated with higher likelihood of coronary plaque, more segments involved (SIS ≥4), high CACS, more significant (stenosis ≥50%) plaques, and more calcified plaques. When focusing on more specific plaque features, participants with the lowest DI tertile (DI 0–5) had 23% higher likelihood for non-calcified non-significant stenosis (OR = 1.23, 95% confidence interval (CI) 1.06 to 1.42), 37% higher likelihood for calcified non-significant stenosis (OR = 1.37, 95% CI 1.13 to 1.66), and 67% higher likelihood for non-calcified and significant stenosis (OR = 1.67, 95% CI 1.21 to 2.33) compared to the highest tertile (DI 8–14) (*Figure 2C*).

When focusing on the plaque presence in different segments of the coronary tree, significant associations were found between lower DI intake and increased plaques in both the right coronary and left anterior descending arteries (see Supplementary material online, *Figure S2*). No major associations were found between the DI and significant (stenosis ≥50%) plaques in neither circumflex nor left main arteries.

**Figure 3:**
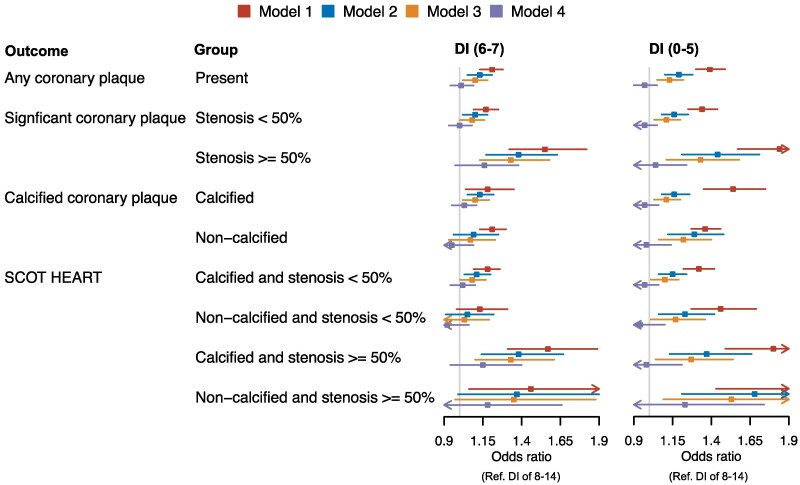
Forest plot showing association of the DI (in tertiles) and plaque phenotypes. ORs and 95% confidence intervals are reported, using subjects with DI of 8–14 as the reference. Model 1 unadjusted. Model 2 is adjusted for age and sex. Model 3 is adjusted for age, sex, and smoking. Model 4 is adjusted for age, sex, smoking, waist circumference, TG, and hypertension. SCOT HEART, plaque phenotypes according to the Scottish COmputed Tomography of the HEART Trial.

Finally, four regression models were tested to ascertain the associations between DI and the various coronary plaque outcomes (*Figure 3*). In the unadjusted model 1, the lowest DI tertile (DI 0–5) as well as the middle DI tertile (DI 6–7) were associated with higher odds of any coronary plaque (*Figure 3*) with up to 39% higher odds of any coronary plaque, up to 84% higher odds for significant stenosis, up to 36% higher odds for non-calcified plaques and with up to 97% higher odds for non-calcified and significant stenosis. The lowest and middle tertiles of the DI were associated with higher presence of any plaque, calcified plaque, and known high-risk plaque features (model 3, adjusted for age, sex, and smoking). When further adjustments were performed in model 4, including potential mediators WC, TG, and hypertension (see Supplementary material online, *Table S4*), or model 5 (including all accessible variables, Supplementary material online, *Table S5*), statistically significant associations between the DI and any outcomes were not found.

### 3.3 Mediators of the association between the DI and coronary atherosclerosis

Our results suggest that WC, hypertension, and TG are potential mediators (see Supplementary material online, *Tables S6*-*S8*) in accordance with the literature.^26^ The largest mediator was WC accounting for 34.4% to 56.7% of plaque phenotype differences followed by TG (35.1% to 39.8%) and hypertension (20.9% to 32.1%) (see Supplementary material online, *Figure S3*).

## 4. Discussion

In this large cross-sectional population-based study, with a novel approach linking coronary imaging by state-of-the-art CCTA and dietary patterns, it was found that a fibre-rich diet including low intake of red and processed meat is associated with lower circulating hsCRP and more relevantly, with more stable coronary plaque phenotypes. Corresponding associations were found for the presence of plaque, involved segments, CACS, and significance of stenosis. Therefore, this data indicates that the association between a high DI and lower hsCRP may be due to anti-inflammatory components. Thus, a dietary pattern high in fibre-rich, plant-based foods and low in red and processed meat, chips, and sugar-sweetened beverages is associated with reduced low-grade inflammation and lower prevalence of coronary plaques with high-risk features. The association between hsCRP and the DI confirms and validates the use of this index, originally associated with inflammation in its primary publication by Kaluza *et al*.^14^ However, hsCRP contributes relatively poorly to either diet or plaque phenotypes comparing to the other studied factors.

This is the first large study on middle-aged men and women, without prior coronary heart disease, who reported their dietary habits, while undergoing extensive cardiovascular risk profiling and coronary tree assessment with CCTA. Two previously reported cross-sectional ultrasound studies of healthy individuals found lower prevalence of carotid plaque and intima-media thickness with higher intake of linolenic acid^27^ and lower CACS with higher intake of magnesium.^28^ In another cross-sectional study of coronary patients using invasive optical coherence tomography, high intake of vegetables, fruit, fibre, folate, and vitamin C was protective, whereas salt and sodium intake were associated with plaque vulnerability.^29^ In the randomized, controlled CORDIOPREV-trial including healthy subjects, a Mediterranean diet led to fewer and lower carotid plaques by ultrasound after 5 and 7 years,^30^ while no effect on carotid plaque regression was found after an intensive lifestyle intervention in patients with coronary heart disease.^31^

Our plaque assessment was not limited to the presence/absence of plaque, degree of stenosis or even CACS,^32^ but rather extended to a more granular plaque phenotype, including the adverse or high-risk plaque feature of low attenuation (no calcification).^23^ The presence of plaques, especially if significant (≥50% stenosis), with a high CACS^33^ and more segments involved,^32^ is well-known to be deleterious. However, CCTA provided better prognostic discrimination compared to CACS in patients with symptoms,^34^ as it allows a detailed plaque feature evaluation. Several adverse or high-risk plaque features have been proposed,^11,35,36^ such as outward remodelling and low attenuation^23^ but our study only assessed low attenuation. Comparing patients with normal coronaries to patients with significant obstructive disease and high-risk plaque features yielded a > 10-fold increase in the rate of coronary events at 5 years.^11^ Our group with non-calcified and ≥50% stenosis matches this particularly high-risk group identified by Williams *et al*.^11^ Subjects reporting diets with low DI had 22% to 97% higher odds (depending on the adjustment model used) of having these plaques, supporting the association between dietary intake and plaque features for the first time in a cohort of healthy individuals derived from a randomly selected population sample.

In age- and sex-adjusted models, WC was the largest potential mediator in the association between DI and plaque presence. This agrees with obesity, particularly abdominal obesity, being associated with CVD.^1,6^ Results from model 4 suggest that the effect of diet on plaque phenotypes is mediated by WC, hypertension and TG. Dietary patterns associated with obesity and large WC include low intake of vegetables and fruits, and high intake of red and processed meat and sugar-sweetened beverages^37,38^ and with high energy- and fat-intake.^39^ In addition, dietary patterns are interrelated with several adverse lifestyle habits including smoking, alcohol consumption, and low physical activity, which in turn relate to many cardiovascular risk factors present in our extensive list, that were themselves associated with the DI, in accordance with previous literature,^29–31^ possibly explaining the significance loss in the broadly adjusted models. The inclusion of education in the model resulted in weaker associations, however, it cannot be established whether the well-known link between low education and coronary disease might be mediated by diet or by other unmeasured characteristics that may vary between education levels.

A major strength of this study was that it was based on a random sample of middle-aged people without established CVD. Thus, the results can be generalizable to a broad population. Furthermore, coronary plaques were assessed by state-of-the-art technology, going beyond measuring the degree of stenosis to analysing specific plaque features. The association with hsCRP that we found strengthens the method and results. Also, the DI is consistent with other well-known dietary indices regarding included foods.^5^ Overall, the use of an index can be seen either as a limitation, as it suggests only a global pattern of diet, or as a strength, as it measures foods that are consumed in patterns, not as single items.

An important study limitation is that the observational, cross-sectional study design hampers causal inference since both residual confounding and reverse causation may explain the observed associations. Furthermore, as with most studies concerning dietary intake and based on questionnaires, the data requires dietary recall by the subjects, which causes some degree of uncertainty and risk for misreporting food- and beverage-consumption indicated by the low energy intake. To mitigate erroneous recalls, a validated questionnaire was used.^21,40^ Another limitation is, that we do not have information on participants ethnicity, but only the information of whether the subject was born in Sweden or not. The differing associations between the DI and the various coronary arteries were novel, possibly related to the relatively small number of plaques found (see Supplementary material online, *Table S9*), and complex to interpret with certainty, so this may warrant further larger studies for clarification. Another limitation is the grouping of different outcomes which could lead to smaller effective sizes, impacting the statistical power of the findings. Still, it is worth noting that the appreciable effect sizes were accompanied by narrow confidence intervals. To strengthen the validity and generalizability of our findings, larger sample sizes and other study designs are needed for future studies.

### 4.1 Conclusion

Using CCTA, a state-of-the-art technology, in a large cohort derived from a random population sample, we demonstrated a novel association between a dietary pattern characterized by intake of foods with anti-inflammatory properties and coronary plaque features. Subjects reporting a low DI—marked by low intake of fibre-rich foods and high intake of red and processed meats—were more likely to exhibit coronary plaques with high-risk features (32% to 97% unadjusted; 15% to 68% after age and sex adjustment). These individuals also tended to have a greater overall plaque burden, affecting more coronary segments, with higher calcification and more severe stenosis. Importantly, waist circumference, TG levels, and hypertension emerged as potential mediators, suggesting that the adverse effects of the dietary pattern on coronary health may be partially driven through these interconnected metabolic and hemodynamic pathways. Finally, although the clinical implications of our findings need to be studied further, the association between diet and coronary plaque features do support international dietary guidelines that advocate for plant-based diet being cardioprotective, as well as disease-specific dietary advice in clinical praxis.

Translational perspective This study, based on a large cohort free of clinical cardiovascular disease showed an association between coronary plaques features assessed with CCTA and a dietary pattern assessed by a DI. The findings suggest that the diet poor in fibre and rich in red meat is associated with high-risk coronary atherosclerotic plaque features beyond the degree of stenosis but considered important to develop cardiovascular events. These results stress the importance of cardioprotective actions such as advice of plant-based, high-fibre diets as part of the clinical praxis.

## Supplementary Material

cvaf088_Supplementary_Data

## Data Availability

Swedish CardioPulmonary bioImage Study (SCAPIS) has been approved by the Swedish Ethical Review Authority (DNR 2010-228-31M and DNR 2017/183-31). Access to the SCAPIS data may be granted upon application to the SCAPIS Data Access Board (https://www.scapis.org/data-access/), provided that ethical approval has been obtained from the Swedish Ethical Review Authority.
