# Supplementary material for: Low-fibre diet is associated with high-risk coronary plaque features
Source: Cardiovasc Res. 2025 Jun 16;121(8):1204–14. doi: 10.1093/cvr/cvaf088 (PMC12310279; doi:10.1093/cvr/cvaf088)
Supplement: cvaf088_Supplementary_Data [file cvaf088_supplementary_data.zip › Supplemental_material_clean.pdf]

Low-fiber Diet Is Associated with High-Risk Coronary Plaque Features

Ingrid Larsson[a, b,] *Jiangming Sun[c,] *, Shafqat Ahmad[d, e, f], Göran Bergström[a, g], Carl-Johan Carlhäll[h, i,], Kerstin Cederlund[j], Isabel Drake[k], Jan E Engvall[h, i], Mats Eriksson[l], Henrik Hagström[m], Tomas Jernberg[n], Tanja Kero[o, p,] , Krister Lindmark[n, q], Maria Mannila[r], Marju Orho-Melander[k], Araz Rawshani[a, s], Ulf Risérus[t], Annika Rosengren[a, u], Mats Ryberg[q], Caroline Schmidt[a], Emily Sonestedt[k], Maria Wennberg[v], Carl Johan Östgren[i, w], Isabel Goncalves[c, x]

[a]*Department of Molecular and Clinical Medicine, Institute of Medicine, Sahlgrenska Academy, University of Gothenburg, Gothenburg, Sweden.* [b] *Department of Medicine, Sahlgrenska University Hospital, Gothenburg.* [c] *Cardiovascular Research Translational Studies, Department of Clinical Sciences Malmö, Lund University, Malmö, Sweden.* [d]*Molecular Epidemiology and, Science for Life Laboratory, Department of Medical Sciences, Uppsala University, Uppsala, Sweden.*

[e]*Preventive Medicine Division, Harvard Medical School, Brigham and, Women's Hospital, Boston, USA.* [f]*School of Natural Sciences, Technology and Environmental Studies, Södertörn University, Sweden.* [g]*Clinical Physiology, Sahlgrenska University Hospital, Gothenburg, Sweden.*

[h]*Department of Clinical Physiology in Linköping, and, Department of Health, Medicine and, Caring Sciences, Linköping University, Linköping, Sweden.*

[i]*Center for Medical Image Science and, Visualization, Linköping University, Linköping, Sweden.*

[j]*Department of Clinical Science, Intervention and, Technology, Karolinska Institutet, Stockholm, Sweden.*

[k]*Department of Clinical Sciences in Malmö, Lund University, Malmö, Sweden.*

[l]*Karolinska University Hospital, Stockholm, Sweden.*

[m]*Department of Public Health and, Clinical Medicine, Umeå University, and, Heart Centre, Umeå University Hospital, Umeå, Sweden.*

[n]*Department of Clinical Sciences, Danderyd University Hospital, Karolinska Institutet, Stockholm, Sweden*

[o]*Medical Imaging Centre, Uppsala University Hospital, Uppsala, Sweden.*

[p]*Department of Surgical Sciences/Radiology, Uppsala University, Uppsala, Sweden.*

[q]*Department of Public Health and, Clinical Medicine, Umeå University, Umeå, Sweden.*

[r]*Department of Cardiology, and, Clinical Genetics, Karolinska University Hospital, Stockholm, Sweden.* [s]*Department of Cardiology, Sahlgrenska University Hospital, Gothenburg, Sweden.*

[t]*Clinical Nutrition and, Metabolism, Department of Public Health and, Caring Sciences, Uppsala University, Uppsala, Sweden.*

[u]*Department of Medicine Geriatrics and, Emergency Medicine, Sahlgrenska University Hospital Östra Hospital, Gothenburg, Sweden.*

[v]*Department of Public Health and Clinical Medicine, Section of Sustainable Health, Umeå University, Umeå, Sweden.*

[w]*Department of Health, Medicine and, Caring Sciences, Linköping University, Linköping Sweden.*

[x]*Department of Cardiology, Skåne University Hospital, Malmö, Sweden.*

*Shared first authorship.

Supplemental Materials

**Supplemental Methods**	**4**
**Table S1**	**7**
**Table S2**	**8**
**Table S3**	**10**
**Table S4**	**11**
**Table S5**	**12**
**Table S6**	**13**
**Table S7**	**14**
**Table S8**	**15**
**Table S9**	**16**
**Figure S1**	**17**
**Figure S2**	**18**
**Figure S3**	**19**
**Supplemental References**	**20**

Supplemental Methods

PATIENTS UNDERGOING CCTA

In 2769 of the subjects CCTA was either not performed due to contraindications or unwillingness to participate, or the images were of poor quality, hampering appropriate analysis (**Figure 1A).** 1648 subjects were excluded due to known previous cardiovascular disease and/or presence of stents or coronary-bypass grafts. This led to a total CCTA data for 25 737 subjects (**Figure 1A**).

FOOD FREQUENCY QUESTIONNAIRE AND DIET INDEX (DI)

The MiniMeal-Q (FFQ) was completed once by each participant. The examinations including completions of questionnaires by participants were performed during the whole year except for summer and Christmas holidays. It is very difficult in practice to cover the full diet of a subject. Despite this difficulty as diet is such an important and modifiable aspect for the subjects' health, questionnaires are used. The 16-items diet index used cannot cover the full diet and must be regarded as an approximation of a dietary pattern. The study where the DI (originally named anti-inflammatory diet index, AIDI) was initially constructed was based on a 96-item FFQ questionnaire. In that study, these 16 items were one by one significantly correlated with hsCRP. Eleven of the food items were negatively correlated with hsCRP (anti-inflammatory potential) and 5 food items were positively correlated with hsCRP (pro-inflammatory potential). The two cohorts in which the AIDI was developed were also Swedish and included men and women within the same ages as in SCAPIS, which underlies our choice of questionnaire in this study design. For frequency of intake, a nine-grade scale was used from "2-3 times a month" to "5 times a day". For estimation of portion size on cooked dishes, five photo-options representing different portion sizes were presented. (1) The MiniMeal-Q has been validated on energy intake (n=40 men and, women) against the doubly-labelled water method (r=0.34, 95% CI, -0.04, 0.72), (1) as well as on vegetables and, fruit intake against plasma carotenoids (r=0.50, p< 0.01 for women (n=94) and, r=0.31, p< 0.01 for men (n=96) and, on wheat- and, rye whole-grains against alkylresorcinols (r=0.25, p<0.05 for women (n=92) and, r=0.20, n.s. for men (n=93)) (2).

Implausible values of reported daily energy intake were excluded, for women: <500 and, >5000 kcal per day and, for men: <550 and, >6000 kcal per day (3).

CORONARY ATHEROSCLEROTIC PLAQUES ASSESSMENT

Briefly, for coronary artery calcium score, non-contrast images were obtained using ECG-gated CT imaging at 120 kV. CCTA was performed with a dual source CT scanner equipped with a Stellar Detector (Somatom Definition Flash, Siemens Medical Solutions), with Omnipaque® 325 mg I/kg used as contrast medium (350 mg I/mL; GE Healthcare). For assessment of coronary artery disease, the eighteen coronary segment model was used.

MEDIATION ANALYSIS

According to Baron and Kenny criteria, mediation analysis was performed by 3 steps (4). In step 1, association between the diet index (DI), and plaque phenotypes accounting for age and, sex was examined. As shown in Figure 3, DI is a significant predictor of plaque phenotypes. In step 2, linear regressions were conducted to test whether DI were associated with potential mediators respectively for waist circumference, hypertension and, triglycerides adjusting for age, sex and smoking. In step 3, DI and, respective mediators of waist circumference, hypertension and triglycerides were included in the model to inspect their associations with plaque phenotypes using age, sex and smoking as covariates. Details from the mediation analysis are shown in Tables S6-S8.

A multiple mediation analysis was also conducted to compare importance of waist, hypertension and, triglyceride in mediating the DI-plaque phenotypes associations. Multiple mediation analyses were implemented using R package mma (version 10.6-1) which could consider correlations between mediators. Age, sex and smoking were covariates.

ASSOCIATION BETWEEN DI AND CORONARY ATHEROSCLEROSIS IN SEGMENTS

When focusing on the plaque presence in different segments of the coronary tree, significant associations were found between DI and plaques in the right coronary and left anterior descending arteries (**Figure S2**). No major associations were found between DI and significant (stenosis ≥50%) plaques in the circumflex nor left main arteries.

Concerning the plaque distribution along the coronary tree, the right coronary artery and, left anterior descending artery emerged as particularly interesting, also in association with DI, which connects to these being some of the most common vessels for plaque presence (5).

Table S1. Description of cardiovascular risk factors for those included in the analyses.

Risk factor	Definition	Type of data
**Diabetes**	Fasting glucose ≥6.1 mmol/L and, HbA1c <48 mmol/mol or fasting glucose ≥7.0 mmol/L or HbA1c ≥48 mmol/mol	Medical history interview, self-reported and, laboratory results
**Familiy history of MI or stroke, subject's parent or sibling**		Self-reported
**Hyperlipidemia**		Self-reported or statin use
**Hypertension**	>140 systolic or >90 diastolic	Measurements or self-reported medication use
**Obesity**	BMI ≥30.0 kg/m^2	Height and weight are measured and, BMI is calculated
**Smoking**	All tobacco products including snuff	Self-reported
**Waist circumference**		Measurements

Table S2. Characteristics of the SCAPIS (Swedish CArdioPulmonary BioImage Study) population in relation to presence or absence of coronary plaque. All variables are given in number (%) or median (interquartile range), if not stated otherwise.

	Total (n=24079)	Absence of coronary plaque (n=14245)	Presence of coronary plaque (n=9834)	p-value
**Coronary atherosclerotic burden**				
**Calcified coronary plaque**				< 0.001
No plaque	14245 (59.2%)	14245 (100.0%)	0 (0.0%)	
Plaque calcified	8058 (33.5%)	0 (0.0%)	8058 (8.9%)	
Plaque non-calcified	1776 (7.4%)	0 (0.0%)	1776 (18.1%)	
**Significant coronary plaque**				< 0.001
Stenosis ≥50%	1234 (5.1%)	0 (0.0%)	1234 (12.5%)	
Stenosis <50%	8600 (35.7%)	0 (0.0%)	8600 (87.5%)	
No plaque	14245 (59.2%)	14245 (100.0%)	0 (0.0%)	
**SIS (≥4 segments)**				< 0.001
No	22025 (91.5%)	14245 (100.0%)	7780 (79.1%)	
Yes	2054 (8.5%)	0 (0.0%)	2054 (20.9%)	
**Total CACS**				< 0.001
>100	2732 (11.4%)	64 (0.5%)	2668 (27.4%)	
≤100	21185 (88.6%)	14102 (99.5%)	7083 (72.6%)	
**SCOT HEART**				< 0.001
No plaque	14245 (59.2%)	14245 (100.0%)	0 (0.0%)	
Calcified and stenosis <50%	7125 (29.6%)	0 (0.0%)	7125 (72.5%)	
Non-calcified and stenosis <50%	1475 (6.1%)	0 (0.0%)	1475 (15.0%)	
Calcified and stenosis≥50%	933 (3.9%)	0 (0.0%)	933 (9.5%)	
Non-calcified and stenosis≥50%	301 (1.3%)	0 (0.0%)	301 (3.1%)	
**Risk factors**				
**Sex**				< 0.001
**Women**	12340 (51.2%)	8886 (62.4%)	3454 (35.1%	
**Men**	11739 (48.8%)	5359 (37.6%)	6380 (64.9%)	
**Age (years)**	57.1 (53.5, 61.0)	56.1 (52.8, 60.1)	58.7 (55.5, 61.9)	< 0.001
**hsCRP (mg/L)**	1.0 (0.60, 2.1)	1.0 (0.60, 2.0)	1.1 (0.60, 2.3)	< 0.001
**Family history of MI or stroke, subject's parent or sibling**				< 0.001
No	12859 (55.9%)	7992 (58.4%)	4867 (52.2%)	
Yes	10159 (44.1%)	5701 (41.6%)	4458 (47.8%)	
**Hyperlipidemia or statin use**				< 0.001
No	21787 (90.5%)	13383 (93.9%)	8404 (85.5%)	
Yes	2292 (9.5%)	862 (6.1%)	1430 (14.5%)	
**Hypertension, doctor-diagnosed or self-reported**				< 0.001
No	18592 (79.8%)	11745 (84.9%)	6847 (72.2%)	
Yes	4716 (20.2%)	2084 (15.1%)	2632 (27.8%)	
**Diabetes**				< 0.001
No	22556 (93.8%)	13633 (95.8%)	8923 (90.8%)	
Yes	1499 (6.2%)	596 (4.2%)	903 (9.2%)	
**Waist Circumference (cm)**	94.0 (85.0, 102.0)	91.0 (83.0, 100.0)	97.0 (89.0, 105.0)	< 0.001
**Smoking, snuff or nicotine products use**				< 0.001
Current	5466 (23.1%)	2718 (19.4%)	2748 (28.5%)	
Ex-smoker	7567 (32.0%)	4306 (30.7%)	3261 (33.9%)	
Never	10627 (44.9%)	7005 (49.9%)	3622 (37.6%)	
**MVPA, minutes per day**	52.6 (36.3, 72.1)	53.2 (37.2, 72.4)	51.5 (34.7, 71.7)	< 0.001
**Stressed or feelings of sadness/depression in the past 1 year**				< 0.001

**Less stressed and, no feelings of sadness/depression**	14901 (64.4%)	8644 (62.8%)	6257 (66.8%)	
**Stressed, or feelings of sadness/depression**	8246 (35.6%)	5130 (37.2%)	3116 (33.2%)	
**Quality of sleep**				0.883
Badly - Very Badly	3732 (16.0%)	2215 (16.0%)	1517 (16.1%)	
Rather well - Very well	19571 (84.0%)	11641 (84.0%)	7930 (83.9%)	
**Hours of sleep per night**				< 0.001
7 hours	10417 (44.8%)	6299 (45.6%)	4118 (43.7%)	
≤6 hours	8402 (36.1%)	4838 (35.0%)	3564 (37.8%)	
≥8 hours	4424 (19.0%)	2679 (19.4%)	1745 (18.5%)	
**Sleep apnea treated**				< 0.001
No	22630 (97.1%)	13540 (97.9%)	9090 (96.0%)	
Yes	664 (2.9%)	287 (2.1%)	377 (4.0%)	
**Education**				< 0.001
**Undergraduate degree or above**	10953 (46.6%)	6831 (49.0%)	4122 (43.1%)	
**Up to upper secondary school or equivalent**	12569(53.4%)	7122 (51.0%)	5447 (56.9%)	
**Born in Sweden**				0.0127
**No**	3511 (14.9%)	2017 (14.4%)	1494 (15.6%)	
**Yes**	20027 (85.1%)	11957 (85.6%)	8070 (84.3%)	
**Employment**				< 0.001
**No**	3284 (14.0%)	1682 (12.1%)	1602 (16.8%)	
**Yes**	20177 (86.0%)	12245 (87.9%)	7932 (83.2%)	
**Cardiometabolic and renal blood markers**				
**TG (fasting only, mmol/L)**	1.0 (0.80, 1.4)	1.0 (0,70, 1.3)	1.1 (0.80, 1.6)	< 0.001
**Cholesterol (mmol/L)**	5.5 (4.8, 6.2)	5.4 (4.8, 6.1)	5.5 (4.9, 6.2)	< 0.001
**HDL (mmol/L)**	1.6 (1.3, 1.9)	1.6 (1.3, 2.0)	1.5 (1.2, 1.8)	< 0.001
**LDL (mmol/L)**	3.4 (2.8, 4.1)	3.3 (2.8, 4.0)	3.6 (2.9, 4.2)	< 0.001
**eGFR (mL/min per 1.73 m2)**	99.9 (90.0, 104.0)	101.4 (92.8, 105.9)	97.7 (86.9, 103.2)	< 0.001
**Dietary variables**				
**DI**				< 0.001
T1 (0-5)	8344 (34.7%)	4651 (32.7%)	3693 (37.6%)	
T2 (6-7)	9596 (39.9%)	5684 (39.9%)	3912 (39.8%)	
T3 (8-14)	6139 (25.5%)	3910 (27.4%)	2229 (22.7%)	
**Energy intake (kcal/day)**	1585 (1240, 2035)	1572 (1232, 2005)	1604 (1253, 2082)	< 0.001
**Alcohol (g/day)**	6.0 (2.0, 10.9)	5.3 (1.8, 9.8)	6.9 (2.6, 12.3)	< 0.001

P-values are from ANOVA for continuous variables or Chi-squared test for categorical variables.

DI: diet index; CACS: Coronary artery calcium score; MVPA: Moderate-vigorous-intensity physical activity; T1: lowest tertile; T2: middle tertile; T3: highest tertile; SCOT HEART: plaque phenotypes according to the Scottish COmputed Tomography of the HEART Trial.

Table S3. Diet index (DI) and sex in the association with plaque phenotypes.

Outcome	Category	DI	Odds ratio		P for DI in tertiles and sex interaction
			Women (n=12 340)	Men (n= 11 739)	
Any coronary plaque	Present	6 to 7	1.17 (1.04 to 1.29)	1.06 (0.95 to 1.18)	0.04
		0 to 5	1.30 (1.05 to 1.45)	1.08 (0.97 to 1.20)	
Significant coronary plaque	Stenosis < 50%	6 to 7	1.15 (1.05 to 1.27)	1.02 (0.91 to 1.14)	0.14
		0 to 5	1.28 (1.15 to 1.43)	1.04 (0.93 to 1.16)	
	Stenosis ≥ 50%	6 to 7	1.40 (1.05 to 1.88)	1.31 (1.07 to 1.61)	
		0 to 5	1.56 (1.13 to 2.17)	1.33 (1.09 to 1.64)	
Calcified coronary plaque	Calcified	6 to 7	1.17 (1.06 to 1.29)	1.07 (0.96 to 1.22)	0.15
		0 to 5	1.27 (1.14 to 1.43)	1.06 (0.95 to 1.19)	
	Non-Calcified	6 to 7	1.18 (0.97 to 1.44)	0.99 (0.83 to 1.25)	
		0 to 5	1.45 (1.17 to 1.80)	1.15 (0.96 to 1.38)	
SCOT HEART	Calcified and stenosis <50%	6 to 7	1.15 (1.04 to 1.28)	1.04 (0.92 to 1.17)	0.34
		0 to 5	1.25 (1.12 to 1.41)	1.04 (0.93 to 1.17)	
	Non-calcified and stenosis <50%	6 to 7	1.16 (0.93 to 1.43)	0.93 (0.77 to 1.14)	
		0 to 5	1.45 (1.15 to 1.82)	1.06 (0.87 to 1.29)	
	Calcified and stenosis ≥ 50%	6 to 7	1.44 (1.01 to 2.05)	1.30 (1.03 to 1.63)	
		0 to 5	1.63 (1.10 to 2.40)	1.24 (0.99 to 1.56)	
	Non-calcified and stenosis ≥ 50%	6 to 7	1.33 (0.80 to 2.23)	1.36 (0.89 to 2.09)	
		0 to 5	1.44 (0.80 to 2.57)	1.71 (1.13 to 2.59)	

Adjusting for age, odds ratios and 95% confidence intervals are reported, using subjects with DI of 8-14 as the reference. The p-value (P) for DI and sex interaction is obtained from a chi-square test in ANOVA, using the model adjusted for age. SCOT HEART: plaque phenotypes according to the Scottish COmputed Tomography of the HEART Trial

Table S4. P for trend demonstrating whether there is a potential trend across the diet index in tertiles in the association with plaque phenotypes.

Outcome	Group	P (trend) from Model 1	P (trend) from Model 2	P (trend) from Model 3	P (trend) from Model 4
**Any coronary plaque**	Present	<2E-16	6.38E-06	0.0016	0.51
**Significant coronary plaque**	Stenosis < 50%	<2E-16	1.30E-04	0.0082	0.44
	Stenosis >= 50%	1.97E-13	3.06E-05	0.0016	0.69
**Calcified plaque**	Calcified	<2e-16	0.00015	0.01	0.5
	Non-Calcified	1.19E-10	0.00027	0.006	0.81
**SCOT HEART**	Calcified, stenosis < 50%	4.77E-13	0.00095	0.026	0.54
	Non-calcified, stenosis < 50%	1.12E-07	0.0059	0.042	0.46
	Calcified, stenosis >= 50%	6.09E-10	0.0016	0.02	0.87
	Non-calcified, stenosis >= 50%	3.00E-05	0.002	0.014	0.25

SCOT HEART: plaque phenotypes according to the Scottish COmputed Tomography of the HEART Trial. Model 1 unadjusted. Model 2 is adjusted for age and, sex. Model 3 is adjusted for age, sex and smoking. Model 4 is adjusted for age, sex, smoking, waist circumference, triglyceride and hypertension.

Table S5. Association of diet index (DI, in tertiles) and plaque phenotypes.

Outcome	Category	N	DI	Odds ratio (Model 5)
**Any coronary plaque**	Present	3912	6 to 7	1.01 (0.94 to 1.10)
		3693	0 to 5	0.99 (0.91 to 1.07)
		p trend (linear)		0.74
**Significant coronary plaque**	Stenosis < 50%	3402	6 to 7	1.00 (0.93 to 1.09)
		3196	0 to 5	0.97 (0.89 to 1.06)
		p trend (linear)		0.54
	Stenosis ≥ 50%	510	6 to 7	1.18 (0.98 to 1.43)
		497	0 to 5	1.13 (0.93 to 1.38)
		p trend (linear)		0.21
**Calcified coronary plaque**	Calcified	3241	6 to 7	1.03 (0.95 to 1.12)
		2979	0 to 5	0.98 (0.90 to 1.07)
		p trend (linear)		0.66
	Non-Calcified	671	6 to 7	0.96 (0.83 to 1.11)
		714	0 to 5	1.01 (0.86 to 1.18)
		p trend (linear)		0.91
**SCOT HEART**	Calcified and stenosis <50%	2848	6 to 7	1.02 (0.94 to 1.12)
		2611	0 to 5	0.97 (0.89 to 1.07)
		p trend (linear)		0.53
	Non-calcified and stenosis <50%	554	6 to 7	0.92 (0.78 to 1.07)
		585	0 to 5	0.95 (0.80 to 1.12)
		p trend (linear)		0.52
	Calcified and stenosis ≥ 50%	393	6 to 7	1.21 (0.97 to 1.50)
		368	0 to 5	1.09 (0.87 to 1.36)
		p trend (linear)		0.58
	Non-calcified and stenosis ≥ 50%	117	6 to 7	1.19 (0.85 to 1.69)
		129	0 to 5	1.25 (0.87 to 1.79)
		p trend (linear)		0.15

Odds ratios and 95% confidence intervals are reported using DI of 8 to 14 as the reference Model 5 is adjusted for age, sex, smoking, waist circumference, triglyceride (TG), high to sensitivity C to reactive protein (hsCRP), estimated glomerular filtration rate (eGFR), moderate to vigorous physical activity (MVPA), sleep hours, alcohol intake, stress feelings, energy intake, education, born in Sweden, employment and, present of hypertension, hyperlipidemia, diabetes, family history of myocardial infarction or stroke.
SCOT HEART: plaque phenotypes according to the Scottish COmputed Tomography of the HEART Trial.

Table S6. Association between DI and respective potential mediators adjusting for age, sex and smoking.

Mediator	DI	Coefficient	95% CI	
			Lower	Upper
**Waist**	6-7	**0.25**	**0.22**	**0.28**
	0-5	**0.47**	**0.44**	**0.50**
**HTN**	6-7	**0.31**	**0.22**	**0.40**
	0-5	**0.58**	**0.49**	**0.67**
**TG**	6-7	**0.15**	**0.12**	**0.18**
	0-5	**0.29**	**0.25**	**0.32**

DI: diet index; CI: confidence interval; HTN: hypertension; TG: Triglyceride.

Table S7. Association between potential mediator and plaque phenotypes adjusting for DI, age, sex and smoking.

Mediator	Outcome	Category	OR	95% CI	
				lower	upper
Waist	Any coronary plaque	Presence	**1.23**	**1.95**	**1.27**
	Significant coronary plaque	Stenosis <50%	**1.22**	**1.18**	**1.26**
		Stenosis ≥50%	**1.39**	**1.30**	**1.49**
	Calcified coronary plaque	Calcified	**1.21**	**1.17**	**1.25**
		Non-calcified	**1.36**	**1.29**	**1.44**
	SCOT HEART	Calcified and stenosis <50%	**1.19**	**1.15**	**1.23**
		Non-calcified and stenosis <50%	**1.37**	**1.29**	**1.46**
		Calcified and stenosis ≥50%	**1.41**	**1.31**	**1.52**
		Non-calcified and stenosis ≥50%	**1.33**	**1.17**	**1.51**
HTN	Any coronary plaque	Presence	**1.90**	**1.77**	**2.04**
	Significant coronary plaque	Stenosis <50%	**1.80**	**1.68**	**1.93**
		Stenosis ≥50%	**2.83**	**2.47**	**3.23**
	Calcified coronary plaque	Calcified	**1.84**	**1.71**	**1.97**
		Non-calcified	**2.21**	**1.97**	**2.48**
	SCOT HEART	Calcified and stenosis <50%	**1.73**	**1.61**	**1.87**
		Non-calcified and stenosis <50%	**2.15**	**1.89**	**2.44**
		Calcified and stenosis ≥50%	**2.89**	**2.49**	**3.37**
		Non-calcified and stenosis ≥50%	**2.63**	**2.04**	**3.38**
TG	Any coronary plaque	Presence	**1.22**	**1.18**	**1.26**
	Significant coronary plaque	Stenosis <50%	**1.20**	**1.17**	**1.24**
		Stenosis ≥50%	**1.32**	**1.26**	**1.39**
	Calcified coronary plaque	Calcified	**1.20**	**1.17**	**1.24**
		Non-calcified	**1.28**	**1.22**	**1.33**
	SCOT HEART	Calcified and stenosis <50%	**1.18**	**1.14**	**1.23**
		Non-calcified and stenosis <50%	**1.29**	**1.23**	**1.35**
		Calcified and stenosis ≥50%	**1.33**	**1.26**	**1.40**
		Non-calcified and stenosis ≥50%	**1.33**	**1.23**	**1.43**

Odds ratio per 1 standard deviation increase of waist or TG are reported. For HTN, odds ratio is reported using patients without hypertension as reference.
DI: diet index; CI: confidence interval; OR: odds ratio. HTN: hypertension; TG: triglyceride; SCOT HEART: plaque phenotypes according to the Scottish COmputed Tomography of the HEART Trial.

Table S8. Association between DI and, plaque phenotypes adjusting for respective mediator, age, sex, and smoking. Odds ratios are reported using DI of 8 to 14 as the reference.

			**Mediator (OR and, 95% CI)**		
**Outcome**	**Category**	**DI**	**Waist**	**HTN**	**TG**
**Any coronary plaque**	**Present**	6 to 7	1.04 (0.97 to 1.11)	1.06 (0.99 to 1.14)	1.07 (0.99 to 1.15)
		0 to 5	1.02 (0.94 to 1.10)	1.06 (0.98 to 1.15)	1.07 (0.99 to 1.16)
**Significant coronary plaque**	**Stenosis < 50%**	6 to 7	1.02 (0.95 to 1.10)	1.04 (0.97 to 1.12)	1.05 (0.97 to 1.13)
		0 to 5	1.01 (0.93 to 1.10)	1.05 (0.97 to 1.14)	1.06 (0.98 to 1.14)
	**Stenosis ≥ 50%**	6 to 7	1.22 (1.03 to 1.45)	1.24 (1.05 to 1.48)	1.28 (1.08 to 1.52)
		0 to 5	1.14 (0.95 to 1.36)	1.18 (0.99 to 1.41)	1.22 (1.03 to 1.46)
**Calcified coronary plaque**	**Calcified**	6 to 7	1.05 (0.98 to 1.14)	1.07 (0.99 to 1.15)	1.08 (1.00 to 1.16)
		0 to 5	1.02 (0.94 to 1.10)	1.05 (0.97 to 1.14)	1.05 (0.97 to 1.14)
	**Non-calcified**	6 to 7	0.99 (0.86 to 1.13)	1.02 (0.89 to 1.17)	1.03 (0.90 to 1.19)
		0 to 5	1.05 (0.91 to 1.21)	1.12 (0.97 to 1.29)	1.14 (0.99 to 1.31)
**SCOT HEART**	**Calcified and stenosis <50%**	6 to 7	1.04 (0.96 to 1.12)	1.05 (0.97 to 1.14)	1.06 (0.98 to 1.15)
		0 to 5	1.01 (0.93 to 1.10)	1.04 (0.96 to 1.14)	1.05 (0.96 to 1.14)
	**Non-calcified and stenosis <50%**	6 to 7	0.95 (0.82 to 1.10)	0.98 (0.85 to 1.14)	1.00 (0.86 to 1.15)
		0 to 5	1.00 (0.86 to 1.17)	1.08 (0.93 to 1.26)	1.09 (0.94 to 1.27)
	**Calcified and stenosis ≥ 50%**	6 to 7	1.21 (1.001 to 1.47)	1.23 (1.01 to 1.50)	1.28 (1.06 to 1.56)
		0 to 5	1.08 (0.88 to 1.32)	1.12 (0.92 to 1.37)	1.17 (0.95 to 1.42)
	**Non-calcified and stenosis ≥ 50%**	6 to 7	1.25 (0.90 to 1.75)	1.28 (0.91 to 1.79)	1.27 (0.91 to 1.78)
		0 to 5	1.34 (0.95 to 1.89)	1.38 (0.98 to 1.94)	1.41 (1.01 to 1.99)

DI: diet index; CI: confidence interval; HTN: hypertension; OR: odds ratio; TG: triglycerides; SCOT HEART: plaque phenotypes according to the Scottish COmputed Tomography of the HEART Trial.

Table S9. Frequency of plaques found in coronary artery segments.

	**Stenosis**	**n**
RCA	No plaque	20051
	<50%	3701
	>=50%	297
Cx	No plaque	20935
	<50%	2879
	>=50%	215
LM	No plaque	21474
	<50%	2191
	>=50%	35
LAD	No plaque	15639
	<50%	7446
	>=50%	940
Cx, LM, LAD and IM	No plaque	14220
	<50%	8760
	>=50%	1097
LM, LAD and IM	No plaque	14593
	<50%	8497
	>=50%	986

RCA: right coronary artery; Cx: circumflex artery; LM: left main coronary artery; LAD: left anterior descending artery; IM: intermediate artery

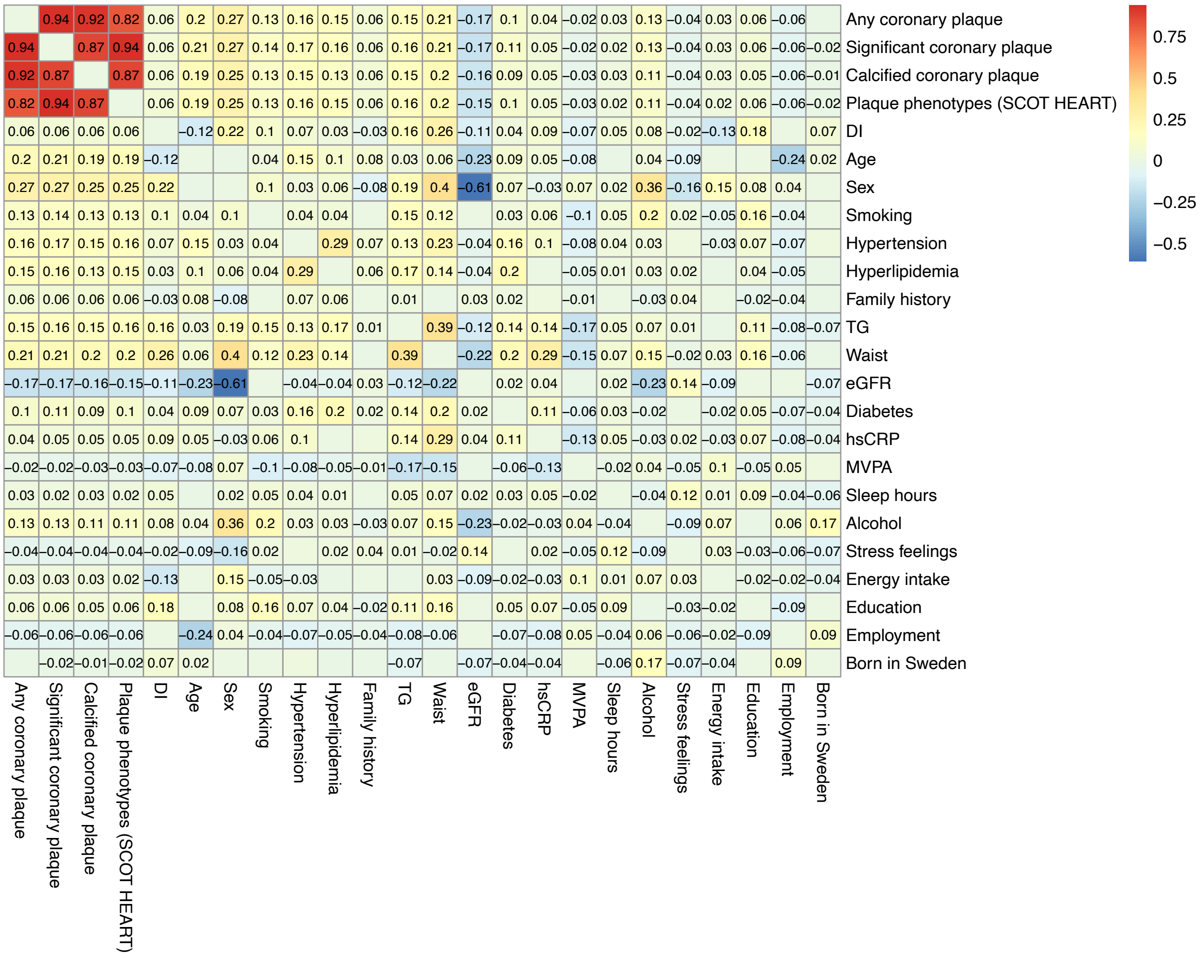

Figure S1. Heatmap showing correlation between variables. 24 079 participants are included in the analyses. Pearson correlation coefficient is displayed if the corresponding p-value is significant (less than 0.05). Significant coronary plaque is encoded as 0 (no plaque), 1 (plaque with stenosis <50%) and 2 (plaque with stenosis ≥ 50%). Calcified coronary plaque is encoded as 0 (no plaque), 1 (calcified plaque) and, 2 (non-calcified plaque). Plaque phenotype (according to SCOT Heart) is encoded as 0 (no plaque), 1 (calcified plaque with stenosis <50%), 2 (non-calcified plaque with stenosis <50%), 3 (calcified plaque with stenosis ≥50%) and 4 (non-calcified plaque with stenosis ≥50%). Sex is encoded as 0 (women) and, 1 (men). TG: triglyceride; eGFR: estimated glomerular filtration rate; DI: diet index; hsCRP: high-sensitivity C-reactive protein; MVPA: moderate vigorous physical activity.

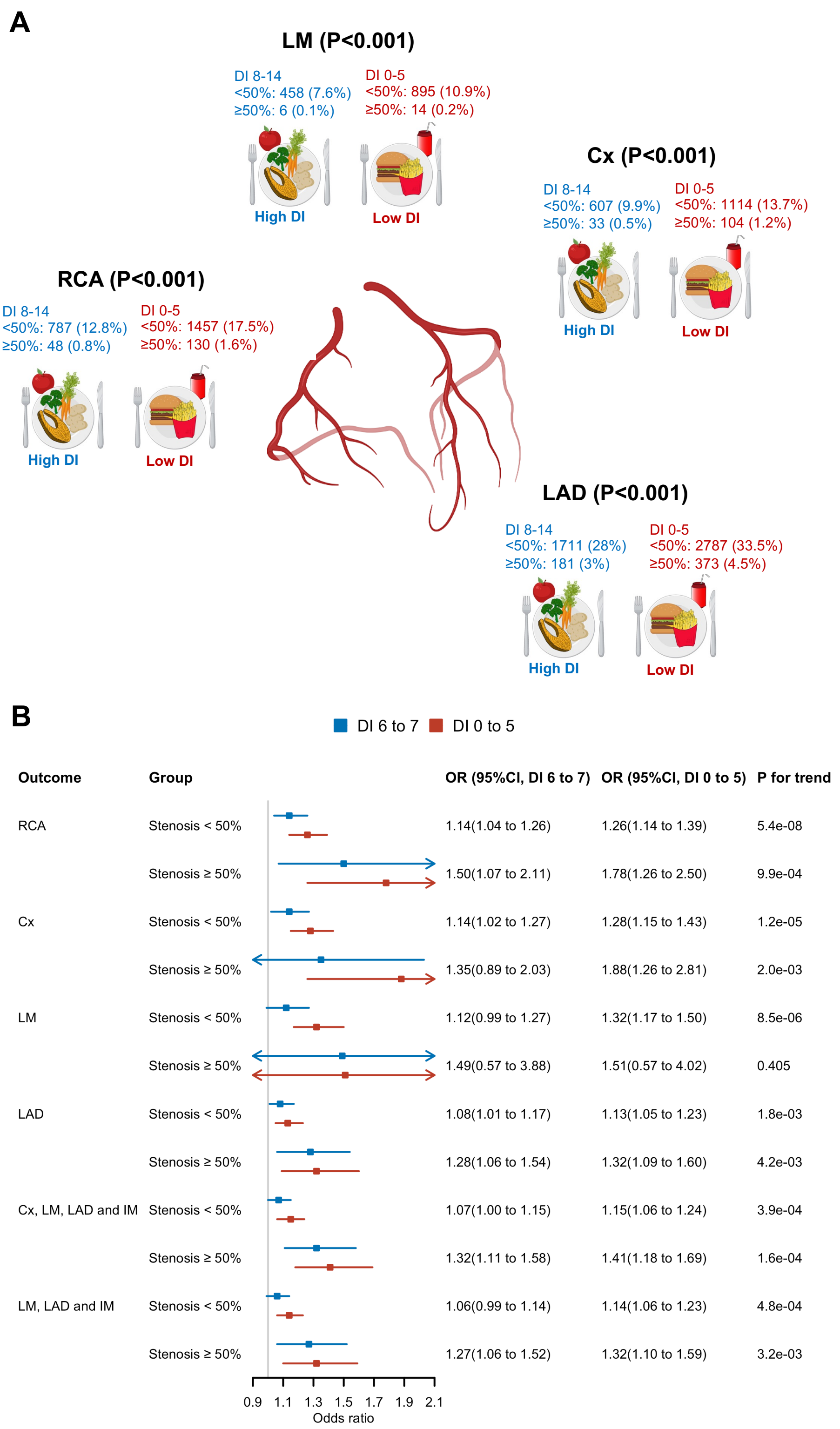

Figure S2. Odds ratios (OR) for association between diet index (DI), and plaque in the coronary tree. 24 079 participants are included in the analyses. **A**) Coronary tree with the number (%) of coronary stenoses <50% or ≥50% in relation to the tertiles of DI. **B**) Forest plot. RCA: right coronary artery (segments 1-4); Cx: circumflex artery (segments 11-16); LM: left main artery (segment 5); LAD: left anterior descending artery (segments 6-10); IM: intermediate branch (segment 17); Cx, LM, LAD and, IM (segments 5-17); LM, LAD and, IM (segments 5-10 and, 17).

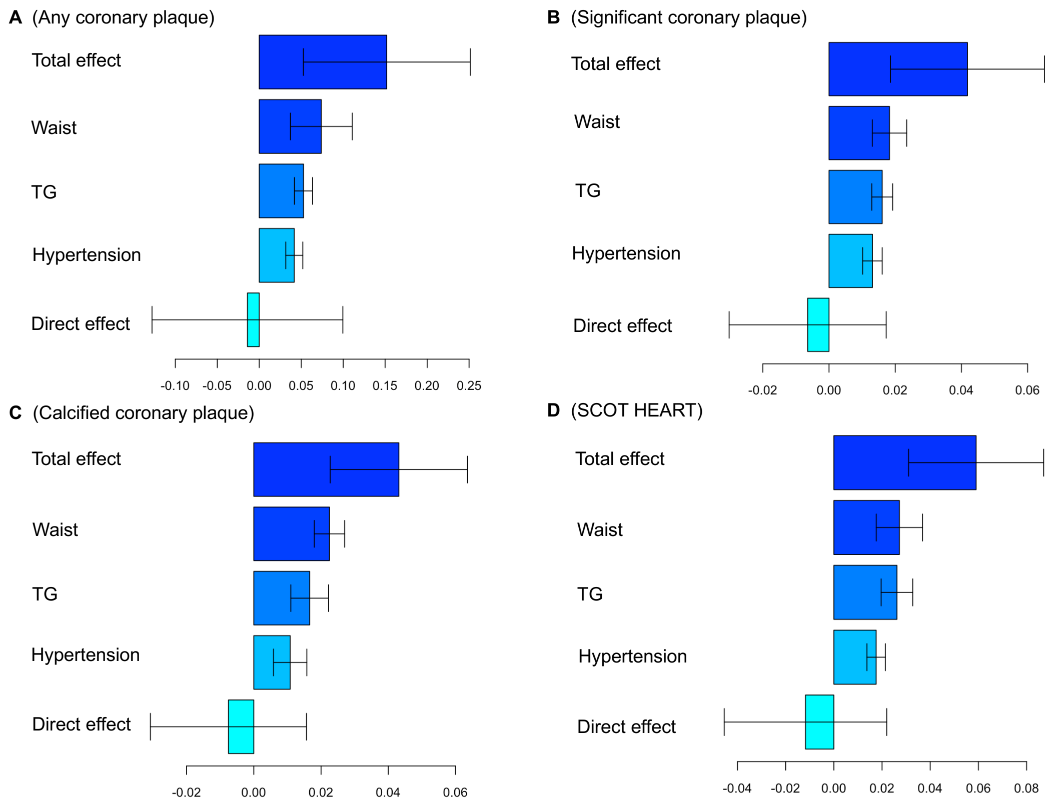

Figure S3. Results from multiple mediation analysis for any coronary plaque. 24 079 participants are included in the analyses. (**A**), significant coronary plaque (**B**), calcified coronary plaque (**C**) and, plaque phenotype (**D**). The estimated mediation effects and, 95% confidence intervals for waist, triglycerides (TG), and hypertension are examined adjusting for age, sex and smoking. Direct effects for diet index on plaque phenotypes is also shown.

Supplemental References

1. Nybacka S, Berteus Forslund H, Wirfalt E, Larsson I, Ericson U, Warensjo Lemming E, et al. Comparison of a web-based food record tool and, a food-frequency questionnaire and, objective validation using the doubly labelled water technique in a Swedish middle-aged population. J Nutr Sci. 2016;5:e39
2. Nybacka S, Lindroos AK, Wirfält E, Leanderson P, Landberg R, Ericson U, et al. Carotenoids and, alkylresorcinols as objective biomarkers of diet quality when assessing the validity of a web-based food record tool and, a food frequency questionnaire in a middle-aged population. BMC Nutrition. 2016;2(53):1-12.)
3. Rhee JJ, Sampson L, Cho E, Hughes MD, Hu FB, Willett WC. Comparison of methods to account for implausible reporting of energy intake in epidemiologic studies. Am J Epidemiol. 2015;181(4):225-33.
4. Baron RM, Kenny DA. The moderator-mediator variable distinction in social psychological research: conceptual, strategic, and, statistical considerations. J Pers Soc Psychol. 1986;51(6):1173-82
5. Bergstrom G, Persson M, Adiels M, Bjornson E, Bonander C, Ahlstrom H, et al. Prevalence of Subclinical Coronary Artery Atherosclerosis in the General Population. Circulation. 2021;144(12):916-29. Nasir K, Cainzos-Achirica M, Valero-Elizondo J, Ali SS, Havistin R, Lakshman S, et al. Coronary Atherosclerosis in an Asymptomatic U.S. Population: Miami Heart Study at Baptist Health South Florida. JACC Cardiovasc Imaging. 2022;15(9):1604-1618
